# Rotablation of a tricky calcified lesion in a tortuous right coronary artery

**DOI:** 10.1007/s12471-019-01363-3

**Published:** 2020-01-17

**Authors:** L. Rijk, S. Hoseyni Guyomi, J. J. Remmen, A. J. M. Oude Ophuis

**Affiliations:** Department of Cardiology, Canisius-Wilhelmina Ziekenhuis, Nijmegen, The Netherlands

A 72-year-old female patient with a medical history of hypertension, diabetes mellitus type 2, chronic obstructive pulmonary disease and coronary artery disease, presented to the outpatient clinic with progressive chest pain. A coronary angiogram revealed significant three-vessel disease. Our regional multidisciplinary Heart Team discussed her case and opted for treatment through multi-vessel percutaneous coronary intervention.

Initial percutaneous coronary intervention of the circumflex artery was successful; revascularisation of the left anterior descending did not succeed because the lesion could not be crossed. Due to persisting chest pain an elective percutaneous coronary intervention of the right coronary artery was performed. The right coronary artery was severely calcified and malformed with a distal functional occlusion (99% stenosis at the time of initial coronary angiography; Fig. 1a).

**Fig. 1:**
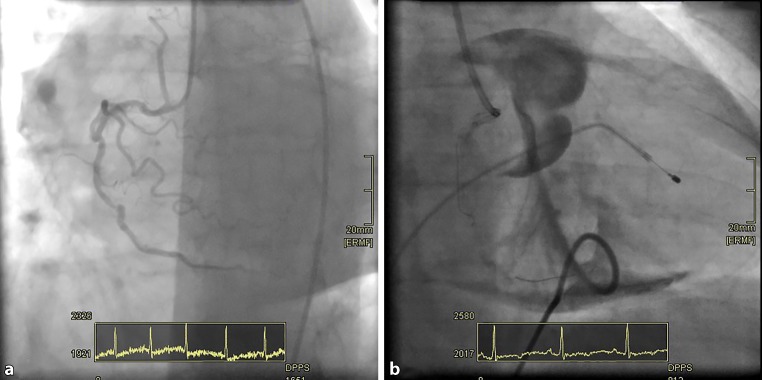
**a** Tortuous and calcified right coronary artery; **b** Angiographic image after rotablation

Access was gained via the right brachial artery using a 7.0 French sheath. After passage with Fielder and Caravel guide wires these were exchanged for a Rotawire, after which rotablation took place (1.25 mm burr, 200.000 RPM).

Fig. 1b shows the angiographic image at the end of the procedure. Describe what can be seen in Fig. 1b, what happened after the start of the rotablation?

## Answer

You will find the answer elsewhere in this issue.

